# Conceptual Ambiguity Surrounding Gamification and Serious Games in Health Care: Literature Review and Development of Game-Based Intervention Reporting Guidelines (GAMING)

**DOI:** 10.2196/30390

**Published:** 2021-09-10

**Authors:** Simon Warsinsky, Manuel Schmidt-Kraepelin, Sascha Rank, Scott Thiebes, Ali Sunyaev

**Affiliations:** 1 Department of Economics and Management Karlsruhe Institute of Technology Karlsruhe Germany

**Keywords:** game-based interventions, gamification, serious games, literature review, reporting guidelines, conceptual ambiguity

## Abstract

**Background:**

In health care, the use of game-based interventions to increase motivation, engagement, and overall sustainability of health behaviors is steadily becoming more common. The most prevalent types of game-based interventions in health care research are gamification and serious games. Various researchers have discussed substantial conceptual differences between these 2 concepts, supported by empirical studies showing differences in the effects on specific health behaviors. However, researchers also frequently report cases in which terms related to these 2 concepts are used ambiguously or even interchangeably. It remains unclear to what extent existing health care research explicitly distinguishes between gamification and serious games and whether it draws on existing conceptual considerations to do so.

**Objective:**

This study aims to address this lack of knowledge by capturing the current state of conceptualizations of gamification and serious games in health care research. Furthermore, we aim to provide tools for researchers to disambiguate the reporting of game-based interventions.

**Methods:**

We used a 2-step research approach. First, we conducted a systematic literature review of 206 studies, published in the *Journal of Medical Internet Research* and its sister journals, containing terms related to gamification, serious games, or both. We analyzed their conceptualizations of gamification and serious games, as well as the distinctions between the two concepts. Second, based on the literature review findings, we developed a set of guidelines for researchers reporting on game-based interventions and evaluated them with a group of 9 experts from the field.

**Results:**

Our results show that less than half of the concept mentions are accompanied by an explicit definition. To distinguish between the 2 concepts, we identified four common approaches: implicit distinction, synonymous use of terms, serious games as a type of gamified system, and distinction based on the full game dimension. Our Game-Based Intervention Reporting Guidelines (GAMING) consist of 25 items grouped into four topics: conceptual focus, contribution, mindfulness about related concepts, and individual concept definitions.

**Conclusions:**

Conceptualizations of gamification and serious games in health care literature are strongly heterogeneous, leading to conceptual ambiguity. Following the GAMING can support authors in rigorous reporting on study results of game-based interventions.

## Introduction

Health interventions that draw on games as their inspirational source (ie, game-based interventions) come with the promise of increasing motivation, engagement, and overall sustainability of health behaviors [1] by enabling new paths of interactions between health care providers and patients [2]. Although games are present in various forms and genres, they generally share four defining traits [3]: a goal players try to achieve, rules that limit how players can achieve the goal, a feedback system telling players how they can achieve the goal, and voluntary participation of the players.

Over time, a myriad of different labels for game-based interventions have emerged, including playification [4], educational games [5], game-based learning [6], active video games [7], fitnessification [8], fitness games [9], exergames or serious exergames [10], cognitive games [11], simulation games [12], games with a purpose [13,14], persuasive information systems [15], and persuasive games [16]. Among these approaches to game-based health interventions, gamification and serious games have emerged as the 2 prevailing concepts [13,17,18]. Both concepts have been reported to be successfully used in various areas of health care, such as promoting healthier lifestyles, supporting rehabilitation processes, or fostering the education of health professionals [19,20]. However, although serious games have been researched long before the proliferation of computer technology and can be traced back to the 1970s [21], the term gamification only became popular around the 2010s [22] and has since then rapidly gained interest from health care researchers and professionals alike [18].

As both concepts have become increasingly established, scholars have developed a variety of definitions for the terms gamification and serious games [23,24]. In addition, several researchers have discussed substantial conceptual differences between gamification and serious games. For example, Liu et al [25] argued that serious games are full-fledged games that are modeled after but independent and separate from real-world systems, whereas gamification can never exist on its own and is always a part of a real-world system that maintains its instrumental functionality. Such conceptual considerations have been supported by empirical studies that showed differences in the effects of gamification and serious games on specific health behaviors [26]. Consistent with the law of parsimony, the differences in conceptual and empirical levels indicate that gamification and serious games are, in fact, 2 distinct concepts that require separate scholarly consideration [27,28]. However, researchers frequently report cases in which terms related to gamification or serious games are used ambiguously (eg, due to a lack of definitions [29,30]) or even interchangeably [31]. Over the course of our own research endeavors, we have also seen first indications that there is an amalgamation of the terms in the form of authors using them interchangeably or ambiguously. The fuzzy use of the 2 terms can yield negative consequences, such as an impediment to cumulative knowledge development [28,31] or the prevention of collaboration between researchers and practitioners [32]. Some of these issues are already visible in the literature. For example, Koivisto and Hamari [33] reported in their well-cited literature review that they had to consider every paper that was labeled as gamification by the authors, despite being aware of and acknowledging conceptual unclarity between the terms gamification and serious games in some research communities. To ensure the feasibility of the review, they had to perform some substantial abstraction, which “has consequently caused some specifics of the studies to be lost” [33]. In health care, negative effects stemming from conceptual unclarity may also ripple through to practice, as it is not uncommon for game-based health interventions to be built around theoretical knowledge [34,35].

Within health care literature, the extent to which existing research explicitly distinguishes between gamification and serious games and whether it draws on existing conceptual considerations to do so remains unclear. A major challenge for authors may also be that they see themselves faced with several different definitions and conceptual differences of gamification and serious games, each operating on a different level of abstraction, focusing on different characteristics. This can lead to contradictory indications and may make it difficult for the authors to reconcile different views [23]. We argue that more practical guidelines for authors to avoid ambiguities between different types of game-based interventions can help achieve a more accurate attribution of cognitive, affective, and behavioral outcomes to either gamification or serious games and ultimately advance the development of effective interventions. In this study, we aim to provide researchers and practitioners with the necessary tools. To develop such tools, it is necessary to capture the status quo of how health care researchers understand, define, and use the terms gamification and serious games and whether they explicitly distinguish between the 2 concepts. Consequently, we formulated the following research questions (RQs):

RQ1: How does extant research conceptualize gamification and serious games in health care?

RQ2: How does extant research distinguish between gamification and serious games in health care?

RQ3: How can authors be guided to avoid conceptual ambiguity when reporting game-based intervention studies?

To answer our RQs, we use a 2-step research design. In step 1, we analyze the current understanding of the terms *gamification* and *serious games* in health care research by reviewing the literature in the *Journal of Medical Internet Research* and its sister journals. By doing so, we provide a comprehensive overview of existing conceptualizations of gamification and serious games in health care (RQ1) and provide rich insights into the nature of the conceptual unclarities surrounding gamification and serious games (RQ2). In step 2, we derive guidelines for authors of game-based intervention studies that may support them in avoiding conceptual ambiguity when reporting their results (RQ3).

## Methods

### Literature Review

To answer the first two RQs, we conducted a systematic web-based database search following the guidelines by Levy and Ellis [36]. We limited the search to journals of JMIR Publications, as the *Journal of Medical Internet Research* itself is one of the leading medical informatics and health care sciences and services journal with an impact factor of 5.43 [37] and a broad area of topics is covered in more than 30 of its sister journals. To identify relevant literature, we used the search tool on the *JMIR* website [38] to search for papers containing terms related to either gamification (search terms: *gamification*, *gamified*, *gamifying*, or *gamify*) or serious games (search terms: *serious AND games*, *serious AND gaming*, or *serious AND game*) in any field. The search was performed on November 12, 2020, and yielded 271 studies, for which we screened their full text to assess their relevance. In doing so, we excluded 65 studies that did not focus on either gamification or serious games but, for example, only mention them in the keywords of the paper [39] or as a possible future research avenue [40]. We presumed that all articles published in a journal of JMIR Publications belong to the research area of health care (ie, are related to research on efforts made to maintain or restore physical, mental, or emotional well-being [41]). Hence, we were left with 206 studies for further analysis. Our data analysis followed a concept-centric approach informed by Webster and Watson [42]. For each relevant study, we conducted a full-text analysis to identify the applied research methods, health care context (based on a study by Stepanovic and Mettler [19]), and applied conceptualizations of the terms gamification or serious games. Regarding the conceptualization of gamification and serious games, we first analyzed whether and, if so, how each of the studies defined the terms. For the purpose of this study, we broadly construed a definition as a phrase that conveys the meaning of a term. Furthermore, we analyzed the inspiration sources of each provided definition (ie, whether they were based on extant literature or self-developed). In addition, we analyzed whether and how the studies differentiated between gamification and serious games. The full results of our concept-centric data analysis, including all 206 papers, can be found in Multimedia Appendix 1 [1,2,10,18,40,43-243].

### Development of Reporting Guidelines

To develop the reporting guidelines, we used a 3-step approach inspired by Moher et al [244]. First, one of the authors generated an initial list of items for inclusion in the checklist based on the insights of the literature review, as well as our personal experience in the field. These items cover both best practices we selected from studies that clearly define and distinguish serious games and gamification, as well as common pitfalls, inconsistencies, and inaccuracies identified within the reviewed literature. The resulting list was grouped into sets of related items. Second, the initial list of items was discussed by all authors in an iterative process to ensure a common understanding. During this step, items were merged, divided, or specified more precisely as required to develop a comprehensive yet concise list of guidelines. In addition, we developed exemplary statements to provide future authors with concrete starting points for reporting game-based interventions. Third, to pilot test our checklist, we sent it to 9 experts in the field of game-based interventions and asked for feedback regarding comprehensibility and completeness. We then consolidated their feedback and incorporated them into guidelines, where feasible. This resulted in adjustments of 6 items (1a, 1b, 2a, 2b, 2c, and 3.1b), as well as the addition of 2 new items (2d and 2e).

## Results

### Conceptualizations of Gamification and Serious Games in Health Care Literature

#### Characteristics of the Included Studies

The reviewed articles were published in the *Journal of Medical Internet Research* and 10 of its sister journals. Table 1 provides an overview of the number of articles published in each journal. In total, 38.8% (80/206) of studies applied quantitative methods, with the most common individual method being intervention trials (67/206, 32.5%). Another 18.9% (39/206) of studies applied qualitative methods, whereas 30% (62/206) of studies were conceptual in nature. The remaining 12.1% (25/206) of studies used mixed methods. Regarding the health care context [19], a majority of studies (99/206, 48.1%) focused on individual lifestyle habits. The most prominent theme in this context is achieving an increase in the physical activity of users, as examined by 7.3% (15/206) of studies [10,43,44]. Chronic disease management and rehabilitation is covered by 38.3% (79/206) of studies, including studies on dementia [45,46], diabetes [47,48], or depression [49,50]. Furthermore, 23.3% (48/206) of studies focused on supporting health professionals and mostly dealt with education on various topics, such as emergency care [51], specific surgical procedures [52,53], or teaching ethics in medicine [54].

**Table 1:** Number of articles mentioning terms related to gamification, serious games, or both for each journal of JMIR Publications (N=206).

Outlet	Gamification, n (%)	Serious games, n (%)	Both, n (%)	Total, n (%)
JMIR Serious Games	21 (10.2)	38 (18.4)	34 (16.5)	93 (45.1)
Journal of Medical Internet Research	11 (5.3)	18 (8.7)	10 (4.9)	39 (18.9)
JMIR mHealth and uHealth	22 (10.7)	1 (0.5)	4 (1.9)	27 (13.1)
JMIR Research Protocols	14 (6.8)	8 (3.9)	3 (1.5)	25 (12.1)
JMIR Mental Health	1 (0.5)	1 (0.5)	6 (2.9)	8 (3.9)
JMIR Formative Research	5 (2.4)	0 (0)	1 (0.5)	6 (2.9)
JMIR Rehabilitation and Assistive Technologies	1 (0.5)	2 (1)	1 (0.5)	4 (1.9)
JMIR Pediatrics and Parenting	1 (0.5)	0 (0)	0 (0)	1 (0.5)
JMIR Human Factors	1 (0.5)	0 (0)	0 (0)	1 (0.5)
JMIR Medical Education	0 (0)	1 (0.5)	0 (0)	1 (0.5)
JMIR Public Health and Surveillance	1 (0.5)	0 (0)	0 (0)	1 (0.5)

**Figure 1:**
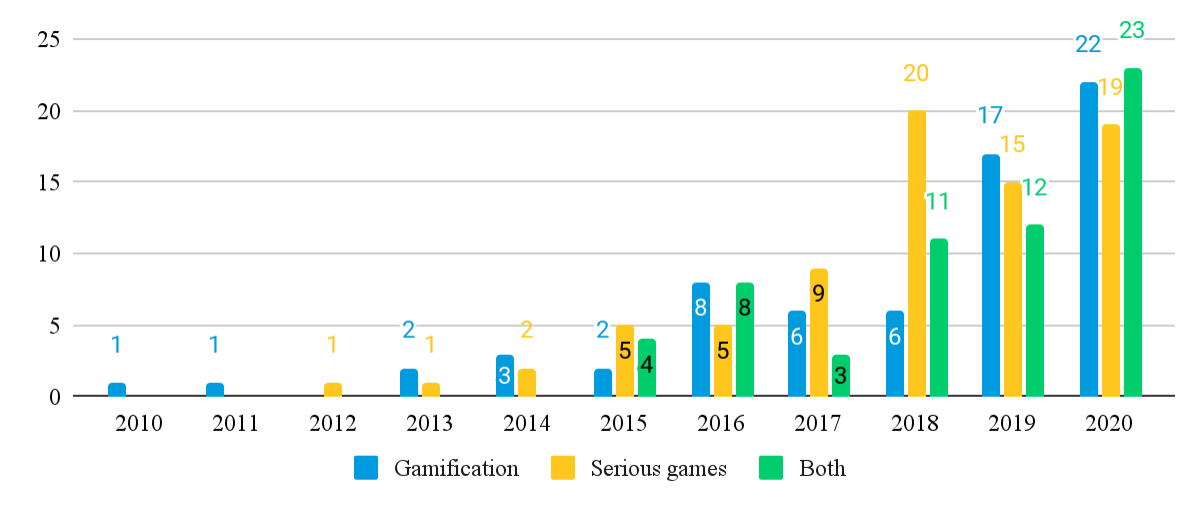
Number of articles in JMIR journals mentioning terms related to gamification, serious games, or both per year.

The 206 studies we reviewed were dated from 2010 to 2020. Across these 206 studies, 137 (66.5%) feature terms related to gamification (*gamification*, *gamified*, *gamifying*, or *gamify*), whereas 128 (62.1%) feature terms related to serious games (*serious AND games*, *serious AND gaming*, or *serious AND game*) in their full text. There is an overlap of 28.6% (59/206) studies that mention terms related to both concepts. Figure 1 shows the distribution of articles that mention terms related to each concept over time. As can be seen in Figure 1, both concepts became increasingly featured in JMIR journals since 2010, with no concept having a clear edge over the other. Out of the 137 studies that feature gamification, 61 (44.5%) explicitly define the term, contrasted by 76 (55.5%) studies that do not. As for serious games, of the 128 studies, 60 (46.9%) that feature the term provide an explicit definition, whereas 68 (53.1%) do not.

#### References to Definitions From Extant Literature

**Figure 2:**
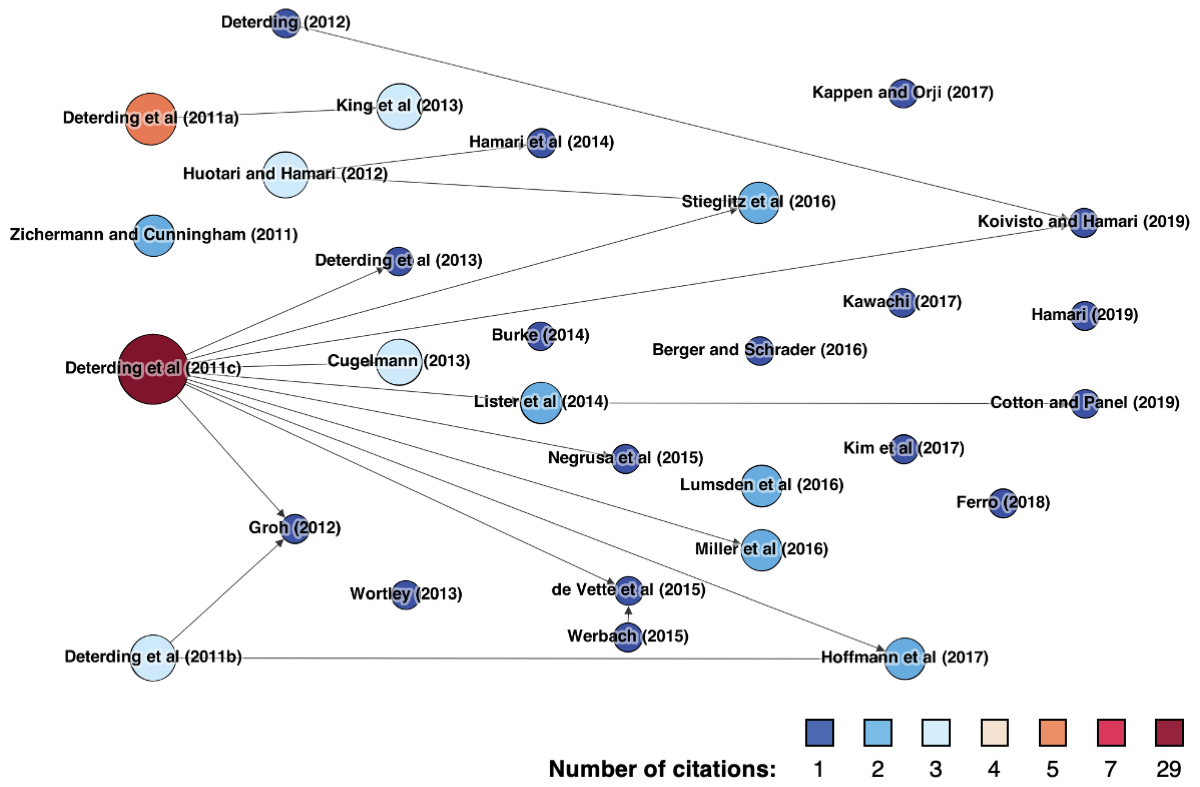
Identified gamification definition papers.

**Figure 3:**
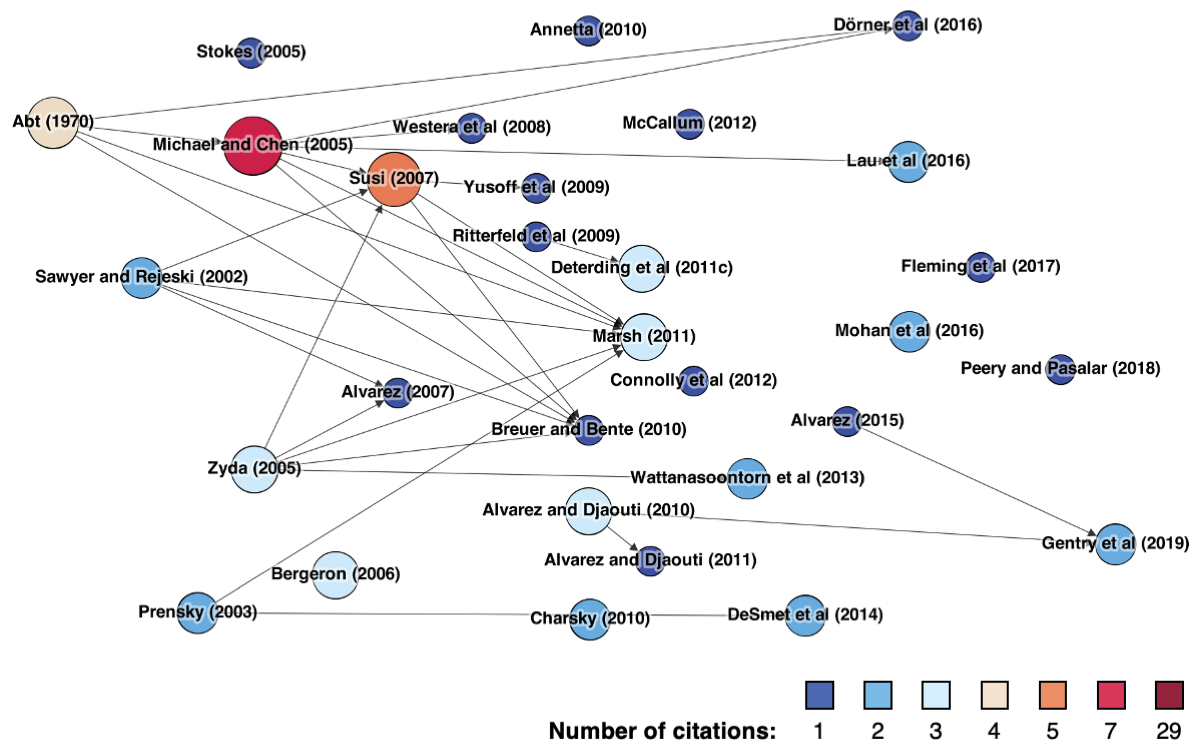
Identified serious games definition papers.

Of the 61 studies that define gamification, a share of 52 (85%) studies explicitly base their definitions on extant literature, whereas 9 (15%) studies provide a self-developed definition. In contrast, out of the 60 studies that define serious games, 50 (83%) explicitly refer to extant literature to do so, whereas 10 (17%) studies do not. In defining gamification, our reviewed papers draw on 29 different sources, whereas for serious games, we found 27 different sources of definitions. Figures 2 and 3 illustrate the different sources our review papers draw on to define gamification or serious games, respectively. Each node represents one such source. The number of times a source has been cited by our review papers is indicated by both the color and size of the corresponding node. An arrow from one node to another indicates that a study draws on another, specifically when defining gamification or serious games.

The most commonly cited source for a gamification definition, cited by 14.1% (29/206) of our reviewed papers, is the seminal paper by Deterding et al [245], who define gamification as “the use of game design elements in nongame contexts.” Considering indirect citations (as indicated by arrows in Figure 2), the percentage value increases to 27.2% (56/206), thus forming the vast majority. Another definition by Deterding et al [246] was used by 2.4% (5/206) of the reviewed papers. Huotari and Hamari [247], Cugelman [1], King et al [248], and Deterding et al [249] are each cited by three (3/206, 1.5%) of the reviewed papers, whereas Zichermann and Cunningham [250], Lister et al [55], Miller et al [251], Lumsden et al [56], Stieglitz et al [252], and Hoffmann et al [57] are each referenced twice (2/206, 1%).

In contrast to gamification, the sources of serious games definitions in our reviewed literature seem to be more fractured and heterogeneous. The most cited paper is that by Michael and Chen [14], cited by 3.4% (7/206) of our reviewed papers. They define serious games as “games that do not have entertainment, enjoyment, or fun as their primary purpose.” In addition to the seven direct citations, Michael and Chen [14] are also cited indirectly 6.3% (13/206) of the time in the definitions of our review papers of serious games (as indicated by arrows in Figure 3). Other prominent sources (based on citation count) include studies by Susi et al [24] (5/206, 2.4%) and Abt [253] (4/206, 1.9%), as well as Deterding et al [245], Zyda [254], Alvarez and Djaouti [255], Bergeron [256], and Marsh [257] (3/206, 1.5%). The remaining sources were cited only once (1/206, 0.5%) or twice (2/206, 1%).

#### Conceptions of Gamification in Health Care

We found that the content of the definitions provided for gamification in the reviewed papers represent several different conceptions. Of the 61 papers defining gamification, 58 (95%) contained only a single definition of the term. Among these articles, 74% (43/58) shared the basic notion of Deterding et al [245,246,249]. They define gamification as either “the use of game design elements in non-game contexts” [245,249] or as an “umbrella term for the use of video game elements (rather than full-fledged games) to improve user experience and user engagement in nongame services and applications” [246]. Both definitions conceptualize gamification as the intentional use of game (design) elements in some kind of nongame artifact. The latter definition, however, explicitly includes the purpose of gamification as improving user experience and user engagement. Similarly, among the 43 papers conceptualizing gamification in line with the basic notion of Deterding et al [245,249], 33 (77%) did not explicitly specify the purpose of gamification. The other 23% (10/43) of papers all specify a variation of engagement, motivation, or both as the purpose of gamification [58-60]. There are also differences regarding the inspiration that gamification draws from games. Although most of the reviewed papers adopt the term game (design) elements from the definition by Deterding et al [245,249], others instead name game (design) techniques [61-64], game principles [65], playful elements [66], game components [67], or game design features [68]. Similarly, the views differed in the artifacts that gamification is applicable to. Instead of nongame contexts, some authors speak of nongame settings [43,69], nongame environments [59], or nongame mechanisms [70]. Others more generally refer to “real world processes or problems” [71], “nongameful or nongamelike situations, services, or tools” [62], or “applications that were not games to begin with” [72].

Another 15 articles provided a single definition that differs from the notion of Deterding et al [245] in a more substantial way. A group of 40% (6/15) of papers specify the nongame context of gamification as either health care [40,73-76] or education [18]. For example, Mendiola et al [75] described gamification as a “feature that offers points, badges, or movement through levels as a health objective is achieved or the more a patient is engaged.” Park and Kim [18] state that “gamification in education applies game elements to an educational context.” Furthermore, 47% (7/15) of articles neither narrowed the context down in a general way nor mentioned a specific context [63,77,78]. For instance, Brown et al [77] refer to gamification as “the application of game design elements to engage and motivate users.” The remaining 13% (2/15) of papers describe gamification as a process to create serious games [79] or focus on gamification as the “overall proliferation of games in culture, society, and technology” [80]. Finally, three papers provide more than one definition of gamification. Among these 3 papers, 2 (67%) [81,82] contrast the aforementioned definition by Deterding et al [245] with an alternative definition that describes gamification as “a process of enhancing a service with affordances for gameful experiences in order to support user’s overall value creation” [247]. Cheng et al [81] argue that the latter definition is more useful in an mHealth context, as it focuses on the goal of gamification instead of its method. Zakaria et al [54] provided various conceptualizations of gamification throughout the article without contrasting them.

#### Conceptions of Serious Games in Health Care

Regarding the content of serious games definitions, we first identified different views on how much serious games draw from games. Most studies simply state that serious games “are games” [83,84], whereas some expand on this by limiting serious games to, for example, “full-blown digital games” [85]. Brown et al [86] used this full game characteristic as the sole basis for their self-developed definition, as they define serious games as “the use of games in their entirety (as opposed to selected elements or individual features of a game).” On the contrary, some authors conceptualize serious games not necessarily as full games but rather as “game-like experiences” [87], “the use of game design elements” [58], or as something that “[uses] specific design principles derived from video games” [88]. Some authors even necessitate the presence of specific game elements in a serious game, such as a challenge [89,90], a responsive narrative [91,92], or interactive elements [93]. Four studies also mention that serious games must be situated in a nongame context [58,86,89,94].

Furthermore, we found that 90% (55/61) of papers that provided a definition of serious games relate to the purpose of a serious game. They do so in any of the following 3 ways. First, 15% (8/55) of studies neutrally state that a serious game needs to have any kind of purpose, also described as a characterizing goal [95]. Second, 40% (22/55) of studies state that a serious game needs to have a specific purpose. For example, Mack et al [96] refer to serious games as “games designed to fulfill a serious purpose by providing education from health professionals via a digital device.” Other mentioned purposes include imparting in a user the skills, knowledge, or attitudes that are applicable in the real world [89], or to improve motivation when completing or addressing complex or bothersome tasks [88]. Third, the remaining 45% (25/55) of studies explicitly exclude specific purposes from being the main purpose of a serious game. This nearly unanimously involved excluding entertainment [87,97] or enjoyment [98] as the main purpose of a serious game. Only Gamito et al [99] pass over entertainment and instead state that “serious games are games designed for other purposes than gaming.”

There were also different views in the reviewed literature on the role of entertainment in serious games. As stated earlier, most studies exclude entertainment as the main purpose of a serious game [87,97]. Nevertheless, some authors still consolidate entertainment as a core part of a serious game by explicitly mentioning that serious games “use entertainment” [100] or “[bring] fun spring from video games” [101] or by referring to serious games as “entertaining games” [102,103]. In contrast, Vilardaga et al [97] refer to serious games as “games not for entertainment,” possibly excluding entertainment as part of a serious game altogether. Furthermore, some authors [95,104,105] seem to consider serious games as an augmented form of (entertainment) games in the sense that they do “not simply [provide] entertainment” [87] or “merely entertain” [106] but rather are a “combination of serious aspects with fun” [101] or “intended to be both entertaining and educational” [103]. The studies by Andrade Ferreira et al [45] and Bindoff et al [107] most vividly represent this view, as they start with entertainment games as a basis, and then outline the differences between serious games and entertainment games (eg, an explicit purpose).

Finally, we identified several aspects that were unique to only one or two definitions. DeSmet et al [103] posit that serious games are “a form of organized play.” Similarly, according to Shiyko et al [94], serious games use playful design strategies. The authors also stated that the term serious games is a synonym for a variety of terms, including gamification [58], applied games [108], games with a purpose [107], and transformational games [45]. In 2 studies, Tuti et al [51,109] posited that serious games have to be playable with a mobile phone. Robert et al [100] remarked that a serious game is a contest played against a computer and thus involves only 1 player.

#### Distinctions

Overall, we identified 59 articles that mention both the terms *gamification* and *serious games* in their full texts. We examined these articles to determine the relationship between the 2 terms used by the authors in each case. To start, 14% (8/59) of articles did not allow for an interpretation of the semantic link between the 2 terms (eg, the terms were used without reference to each other in completely different sections of the article). Within the remaining 86% (51/59) of articles, we identified four groups of articles with distinct approaches: (1) implicit distinction, (2) synonymous use of terms, (3) serious games as a type of gamified system, and (4) distinction based on the full game dimension. The 4 approaches are outlined in detail in the following sections.

The first group contained 29% (15/51) of articles that only implicitly differentiated between the terms *gamification* and *serious games*. The authors of these articles use the 2 terms in relation to each other in such a way that it becomes clear that they differentiate between the two terms without providing any explanations for why and how to do so. Typical examples of this approach include articles that use and differentiate the terms in enumeration. For example, Park and Kim [18] state “[b]efore gamification, educational games, game-based learning, and serious games were applied to the classroom. As gamification was defined, however, it has become preferred among instructors compared with the other techniques.” Similarly, Martin et al [74] write “[I]n all, 2 different persuasive mechanisms—gamification and serious gaming—were proposed.”

The second group contained 10% (5/51) of articles that used the terms gamification and serious games interchangeably. For example, Booth et al [58] state that “[s]erious gaming or gamification has been defined [...] as ‘the use of game design elements in nongame contexts’ for the purposes of engaging learners in solving complex problems.” Although some authors use the terms synonymously without making it a subject of discussion, others show awareness of conceptual ambiguity. For example, Lumsden et al [56] report: “[There is a] lack of coherence in the field [...] partly due to poor definition of terms; for example, the gamelike tasks covered in this review could be described as ‘serious games,’ ‘gamelike’, ‘gamified’, ‘games with a purpose’, ‘gamed-up’, or simply ‘computer based’.”

The third group contains 43% (22/51) of papers that regard serious games as a type of gamified system. Many authors refer to gamification as the process of developing a game-based intervention. For example, Zhang et al [68] stated that “the gamification approaches used included the addition of gaming elements to existing tasks, transformation of a conventional task into a serious game.” Furthermore, while some of these articles focus on serious games as the only possible outcome of gamification, many other authors seem to share the view that other types of game-based interventions can also be the outcome of gamification. For example, Fornasini et al [44] reported that “a recent trend has revealed virtual physical training through exergames and serious games, making gamification an effective tool to motivate.” In this regard, serious games are also sometimes referred to as systems that integrate “gamification principles and gamification strategies” [110]. However, it mostly remains unclear what is meant by these terms and how they differ from other established terms such as game mechanics, game dynamics, and game aesthetics [258].

The last group contains 18% (9/51) of articles that distinguish between gamification and serious games based on whether the system in consideration is regarded as a full game. For example, de Vette et al [111] state that “gamification is defined as the use of elements from games in nongame contexts to improve user experience and engagement without making that system a full game as is the case with serious games.” However, it largely remains unclear what the criteria are that determine whether a system is a full-fledged game or *only* gamified. Some authors try to counteract this by giving examples of gamification and serious games that are as vivid as possible. For example, Vermeir et al [60] state that “an interactive world in which players complete challenges designed to improve physical activity” is a typical example for a serious game, whereas a gamified system could be “a mobile health application that uses points and badges to encourage physical activity.” Nevertheless, these authors also report that, in practice, the actual distinction between the 2 concepts can be blurry and highly subjective because of a lack of clear criteria [60].

### Game-Based Intervention Reporting Guidelines

#### General Remarks About the Guidelines

Through our review, we found that not only are the concepts of gamification and serious games construed in various ways but also their relationship is often fuzzy and unclear. To reduce this conceptual ambiguity, we compiled reporting guidelines consisting of 25 items that are considered essential when reporting studies that deal with game-based interventions and grouped them into 4 topics. In the following sections, we present these items, each accompanied by a description and, where applicable, exemplary statements. A consolidated version that can be used as a quick reference can be found in Multimedia Appendix 2 [245]. A more detailed version including detailed rationales for each individual item may be found in Multimedia Appendix 3 [245].

Game-Based Intervention Reporting Guidelines (GAMING) should be interpreted with some consideration. GAMING are intended to support the reporting of studies on game-based interventions. Although some of the items in GAMING may be beneficial to consider when developing a game-based intervention, our guidelines are not intended to guide the development of game-based interventions per se. GAMING are also intended only for studies that deal with game-based interventions. Accordingly, when we use the term concept in our guidelines, we refer to a type of game-based intervention (eg, gamification, serious games, or exergames). We further differentiated between those concepts that are the thematic focus of the study (henceforth termed *core concepts*) and those that only have auxiliary roles (henceforth termed *related concepts*). An example to clarify this: a study may focus on the development of a physical activity intervention based on the paradigm of serious games (core concept) but still compare its results to gamified physical activity interventions or exergames (related concepts). In the following sections, we briefly introduce the contents of our guidelines and the rationales for including them.

#### Topic 1: Conceptual Focus

The first topic contains items that ensure that the conceptual focus of a study is clear to the authors and readers alike (Table 2).

**Table 2:** Game-Based Intervention Reporting Guidelines—items in topic 1: conceptual focus.

Number and topic	Description	Exemplary or explanatory statements
**1. Conceptual Focus**		
	(a) Decide which concepts (ie, gamification or serious games) best reflect the interventions you want to investigate.	See Multimedia Appendix 4 [17,25,29,31,245,259-261] for existing theoretical distinctions between serious games and gamification.
	(b) Clearly state early in the paper which core concepts (ie, gamification or serious games) you focus on in your study and which criteria the decision for this core concept was based on.	“In this study, we focus on the concept of gamification, because we wanted to bring single game elements into the intervention instead of developing a full-fledged game.”
	(c) Supply only metadata (eg, title and keywords) that corresponds to your core concepts.	For a study developing a physical activity intervention based on gamification: Gamification, gamified intervention.

Ultimately, it should be transparent for the reader, what kind of game-based interventions a study focuses on. A precondition for this is that the authors themselves understand what concepts best reflect the design of the game-based interventions they want to focus on (item 1a). In light of the plethora of existing concepts, as well as the different operationalizations of each, this is by no means a trivial task. Authors may find it beneficial to draw upon concept distinctions from extant literature to establish their conceptual focus. To decide between gamification and serious games, we compiled several distinctions from extant literature that authors may use as decision support (Multimedia Appendix 4). After deciding on their core concepts, the authors should communicate this decision early in their manuscript and include information on how the decision was reached (1b). This allows the reader to comprehend their decisions and grasp the study’s conceptual focus. Furthermore, we suggest that authors should be mindful of the metadata they supply their study with (1c). When reviewing the extant literature, researchers often use metadata to initially assess the conceptual focus of a study and subsequently derive its relevance for their own purposes. Although we acknowledge that broadly diversified metadata increases the visibility of a study, it can also blur the conceptual focus of a study and increase noise for meta-studies or literature reviews.

#### Topic 2: Contribution

The items that we propose for the second topic (Table 3) are intended to clarify the knowledge contribution of a study to both authors and readers.

We believe that there are various research streams proximal to different concepts. What constitutes a research stream can be interpreted in various ways. Broadly, we understand a research stream as a set of studies with similar properties, such as their health care context (eg, all studies that describe the design of gamified physical activity interventions), or their applied methodology (eg, all studies that conduct literature reviews on serious games).

**Table 3:** Game-Based Intervention Reporting Guidelines—items in topic 2: contribution.

Number and topic	Description	Exemplary or explanatory statements
**2. Contribution**		
	(a) Decide which research stream within the focused concepts your study contributes to.	“Our work contributes to a better understanding of how gamification is being applied in real-world mHealth apps.”
	(b) Report which research streams your study contributes to and which criteria the decision for research streams was based on.	“We contribute to a better understanding of the specific game element of leaderboards. We decided for leaderboards, as they are prominently used in mHealth apps to elicit social comparison.”
	(c) Clarify your study's contributions to the chosen research streams, including the boundaries of your study.	“We provide rich insights into the psychological effects of leaderboards on patients when isolated from other game elements. [...] Our insights are about leaderboard specifically and are not necessarily transferable to other social comparison features.”
	(d) Clarify your study’s contributions to solving a problem or need in practice or society.	“The results of our study can support the design and implementation of successful physical activity mHealth apps in practice.”
	(e) Report to which extent observed positive and negative outcomes can be attributed to your game-based interventions. If possible, narrow down the attribution of outcomes based on individual components of your game-based intervention (eg, game elements).	“Participants showed increased motivation, because they were able to compare themselves to others via the leaderboard function. However, this function also caused some participants to feel less competent, thus decreasing their motivation.”

Each research stream comes with its own thematic emphasis and viewing angle. Thus, the transferability of knowledge between different research streams requires care and consciousness. For instance, insights from studies focusing on physical activity are often not transferable to studies focusing on health professionals’ education [19]. Similarly, knowledge about badges is not necessarily transferable to leaderboards, although both approaches may be labeled as gamification. Thus, we think that authors should consciously decide which research streams they contribute to (2a). To facilitate transparency, the authors should communicate their decision to their readers (2b). By also including insights into the decision process (eg, explaining the criteria that shaped the decision for a research stream) in their manuscript, readers can gain a better understanding of a study’s setting and on the generalizability and transferability of its results. After deciding on a research stream, the authors should clarify their study’s contribution to the knowledge base of the research stream, including the boundaries of their study (2c). By doing so, readers can understand how the study contributes to a better understanding of core concepts. Furthermore, the ultimate goal of any intervention is usually to solve a problem in practice (eg, sedentary lifestyles). Accordingly, to foster an understanding of how specific game-based interventions can help to solve these problems, we recommend that the authors clarify their study’s practical contribution (2d). Finally, for an understanding of game-based interventions, it is vital that the outcomes of an intervention are reported and attributed to the chosen type of game-based intervention in an as granular way as possible (2e). In this way, readers can easily contextualize knowledge and understand whether an outcome is, for example, evoked by a specific game element (eg, leaderboards) or connected to a specific affective outcome (eg, envy). We also encourage authors to embrace negative outcomes of their game-based interventions, as these, despite them usually not being the desired outcome of an intervention, can notably contribute to a better understanding of a concept [262].

#### Topic 3: Mindfulness About Related Concepts

Items in the third topic (Table 4) are concerned with ensuring that authors are mindful of the conceptual ambiguities surrounding game-based interventions and possible consequences for their study.

**Table 4:** Game-Based Intervention Reporting Guidelines—items in topic 3: mindfulness about related concepts.

Number and topic		Description	Exemplary or explanatory statements
**3. Mindfulness about related concepts**			
	**3.1. Introduction and use of related concepts**		
		(a) Make efforts to identify possibly related concepts prominent in the context of your study.	Prominent related concepts for gamification in physical activity: Exergames, active video games, fitness games.
		(b) Mention only those related concepts that are substantive for your study.	‘Substantive’ in the sense that a research design necessitates the introduction of a concept. Example: A research design contrasting the effects of two game-based intervention concepts requires the introduction of both concepts.
		(c) Be mindful about nuanced terms in the domain of any introduced concept and use established vocabulary precisely.	Examples: Game design elements, game mechanics, gamification elements, ...
		(d) Avoid using related concepts interchangeably. If you use an umbrella term, specify which terms it comprises and clarify why you introduce it.	“To allow a better readability of the manuscript, we use the term activity games to describe gamified physical activity interventions, serious games for physical activity, as well as exergames.”
	**3.2. Insights from extant literature**		
		(a) Be mindful about conceptual ambiguities when drawing on the literature about game-based interventions.	—^a^
		(b) Do not presume easy transferability of insights from one concept to another.	Example: Drawing on serious games literature for a gamification-based intervention (or vice versa).
		(c) Specify precisely what you draw from the literature and why these insights are applicable to your study.	“Serious games and gamification share that they both center around game elements. Hence, to compile a list of possible game elements for our gamified intervention, we also drew upon serious games literature to widen our scope.”

^a^Not available.

First, we think that studies benefit if authors identify related concepts that are in close proximity to the core concepts (3.1a). Considering any pair of concepts, the boundaries between these closely related concepts will likely be the vaguest, which also makes them the most important boundaries to define to avoid conceptual ambiguities. Authors may assess the “proximity” of 2 concepts based on various contextual factors of an intervention, such as targeted health behavior. For example, in a physical activity context, exergames are a prominent related concept to consider. Similarly, terms with high linguistic proximity are likely to be candidates for related concepts. As Tan et al [72] remarked in their study, the terms *serious games* and *serious gaming*, although ostensibly the same, can be construed as 2 different concepts that describe different classes of game-based interventions (ie, *serious games* describe games specifically designed for the serious purpose of health education, whereas *serious gaming* is the use of any game for said purpose). For each related concept, the authors should also consider the benefits of introducing it in their manuscript. Increasing the number of concepts introduced by nature also increases the possibility of conceptual ambiguity. In our review, we faced anecdotal mentions of gamification or serious games, which were often difficult to interpret regarding the authors’ understanding of the individual concepts and the relationship between the two. Thus, we argue that concepts should only be introduced when they are substantive for understanding a study (3.1b). This also leads to less noise for meta-studies as well as less confusion for readers, as they have to keep track of only substantive concepts. Our guidelines also account for mindfulness regarding conceptual ambiguities in the vocabulary surrounding game-based interventions (3.1c). In game-based interventions, many terms can be quite nuanced or not necessarily correspond to its intuitive meaning. For instance, in games research, the term *game aesthetics* not only describes the artistic value of a game’s visual interface but may also refer to the desirable emotional responses evoked in the player [258]. Accordingly, we suggest being precise while using vocabulary in the domain of any concept and not lightheartedly use or arbitrarily adapt possibly nuanced terms. As there are at least theoretical differences between most concepts, we also highly discourage the interchangeable use of concepts in a manuscript (3.1d). If several concepts are summarized under an umbrella term, this should be done in a transparent way to avoid confusing the reader.

Furthermore, we think that authors should be mindful of the applicability of insights from extant literature to their own research in 2 ways. First, if a researcher finds a study that is labeled as focusing on the same concept that they are trying to investigate, it does not necessarily follow that the researcher and the authors of said study have the same understanding of the concept in question. As our literature review illustrates for gamification and serious games, there are various understandings that are not always reconcilable. Hence, it is advisable to be mindful about conceptual ambiguities when reviewing the extant literature on game-based interventions (3.2a) and ideally challenging the label of each study against one’s own understanding. Second, when transferring knowledge from one concept to another, the authors should carefully consider the possible theoretical and empirical differences in the respective concepts. This aspect is supported by extant research that found several empirical differences with regard to the psychological and behavioral outcomes of gamification and serious games [26]. Such differences can easily impede the transferability of knowledge from one concept to another. Thus, the authors should not presume the easy transferability of knowledge from one concept to another (3.2b). Furthermore, after assessing transferability, authors should precisely specify what knowledge they draw from extant literature and why they think that said knowledge is transferable to their study (3.2c). This empowers the reader to put statements into the context of extant literature and allows a better understanding of how knowledge may be transferable, despite possible conceptual differences.

#### Topic 4: Individual Concept Definitions

The items in the fourth topic (Table 5) focus on aligning the understandings of authors and the reader regarding the introduced concepts.

**Table 5:** Game-Based Intervention Reporting Guidelines—items in topic 4: individual concept definitions.

Number and topic			Description		Exemplary or explanatory statements
**4. Individual concept definitions**					
	**4.1. Definition inspiration**				
		(a) Familiarize yourself with definitions for a concept provided by extant literature.		Reviews can often provide a good overview of different views on a concept.	
		(b) Decide whether a concept definition from extant literature is applicable for your research or if you need a self-developed definition.		Decision criteria: Deficits in extant literature? Incompatibility of own views with literature?	
	**4.2. Definition of concepts**				
		(a) Explicitly define each introduced concept independently in a principal clause. Ideally, justify your choice for a specific definition.		“We define gamification as the use of game design elements in non-game contexts, as this is the most widely applied definition of gamification across disciplines. We define serious games as games whose primary purpose is not entertainment. A game is [...].”	
		(b) Explicitly distinguish each introduced related concept pairwise to at least to your core concepts; better even to all related concepts.		"Gamification differs from serious games in that [...]."	
	**4.3. Definitions from extant literature: if definitions are taken from extant literature...**				
		(a) Make efforts to identify the original source of a definition.		—^a^	
		(b) Include an explicit reference to the source of a definition directly following the definition.		“We define gamification as the use of game-design elements in non-game contexts” [Deterding et al, 2011]; [245]	
	**4.4. Self-developed definitions: if any definition for a concept is self-developed...**				
		(a) Make sure to adhere to good definition design.		Be specific, avoid long sentences, do not repeat the term to be defined in the definition.	
		(b) Clarify from which views your self-developed definition emerged.		“We include only specific game elements of point, badges and leaderboards in our definition of gamification, because [...].”	
	**4.5. Multiple definitions for a single concept: if multiple definitions for a single concept are provided...**				
		(a) State clearly, which definition(s) is (are) applied in the study, and why.		“Gamification can be either defined as [...][exemplary reference 1] or as [...][exemplary reference 2].”; “For the purposes of this study, we follow the view of [exemplary reference 2], because [...].”	
		(b) Apply the chosen definition(s) consistently.		—	

^a^Not available.

They cover individual concept definitions as well as the relationships between concepts. Having chosen that they want to introduce a certain concept, authors should first familiarize themselves with how extant literature defines it (4.1a). They should then decide whether they want to adopt a definition from the extant literature or need a self-developed definition (4.1b). The latter may especially be the case if authors find that there are no definitions for a concept in extant literature, deficits in existing definitions, or if they feel that their view of a concept is not sufficiently covered by any existing definition. Challenging existing definitions can bring vast benefits to the theoretical development of a research stream. In most cases, however, authors are better off when they build on existing definitions, as this may allow better placement within extant research and facilitate the building of cumulative knowledge. In particular, we urge not to *reinvent the wheel* when providing any self-developed definition. For most game-based intervention concepts, multiple prominent definitions exist that together cover a large range of views (eg, the studies by Deterding et al [245] or Huotari and Hamari [247] for gamification and the studies by Michael and Chen [14] or Alvarez and Djaouti [255] for serious games). Hence, authors should always consider first if they want to align their views with those of the extant literature.

Irrespective of the source of a definition, it is vital that authors explicitly define every concept introduced in their study (4.2a) to ensure that readers and authors share a common understanding. A lack of an explicit definition for a concept requires the reader to subjectively appraise the authors’ understanding of it based on context. Throughout our literature review, we found that about half of the mentions of gamification or serious games are not accompanied by an explicit definition and thus require such an appraisal, which is likely error-prone. Furthermore, concepts should not be initially defined based on their boundaries to other concepts but rather in an independent manner. For example, statements such as “exergames are games that differ from serious games in [...]” do not fully reveal an author’s understanding of exergames to readers, but only that they somehow differ from serious games. Defining concepts should be done in a principal clause to ensure that a definition is actually recognized as such. Ideally, authors should also justify their choice for a particular concept definition, as this can provide valuable context to the reader (eg, the knowledge that a particular definition is the most used in a particular discipline). Once all concepts (ie, core concepts and related concepts that are substantive in the sense of 3.1b) are independently defined, the boundaries between concepts can be established (4.2b). Explicitly drawing the boundaries between concepts is the most reliable way to mitigate conceptual ambiguities. Ideally, the boundaries between all possible pairs of introduced concepts are drawn. In cases where this is infeasible (eg, due to a large number of introduced concepts), the boundaries adjacent to the core concepts of a study take priority. The study by Park and Kim [18] is a good example to note the benefits of properly introducing and delineating related concepts. They focus on developing a gamified learning environment, making gamification their core concept. Owing to their proximity to the education context, they identify the related concepts of game-based learning and educational gaming, define them individually, and subsequently sharpen their contribution by stating that gamification is their preferred intervention design for being a more accessible technique for teachers than the other concepts.

If authors decide to adopt definitions from the extant literature, it is important that they establish a clear and unambiguous connection to their respective sources. To do so, researchers should first aim to identify the original source of a definition (4.3a). Readers with domain knowledge are usually able to recognize prominent sources of definitions (especially in the case of seminal papers) and can then immediately attribute a study to a certain view of a concept. Not citing the original source of a definition (ie, indirect citation) obfuscates the original source of a definition and prevents this process. To ensure that a definition can be attributed to its source, the source should be explicitly referenced immediately after the principal clause that contains the definition (4.3b).

Formulating a *good* self-developed definition is a nontrivial task that requires great care. Authors that decide to do so find support in guidelines for a good definition design (4.4a). Although there is no universal way to formulate definitions, authors may consider general guidelines on definition design [263] or take guidance from definition development principles in other areas [264]. Within the context of our study, we particularly emphasize avoiding the use of any part of the term that is defined within the definition of the term itself. For example, when defining serious games as *games with a serious purpose*, the question immediately follows, how *games* and *serious purposes* are defined, essentially leaving the reader none the wiser concerning the meaning of serious games. Whenever authors decide on a self-developed definition of a concept (considering 4.1b), they should also allow the reader to comprehend this decision by clarifying from which view their self-developed definition emerged (4.4b). By fostering an understanding of the decision for a self-developed definition, the reader can also better grasp how a study contributes to an advanced, possibly novel understanding of a concept.

Finally, in some cases, authors may find it beneficial to name several definitions of a single concept, for example, to contrast different views [81,265] or to outline the theoretical development of a concept [112]. To avoid confusing the reader, they should clarify which definition they apply in their study (4.5a) and subsequently apply this definition consistently (4.5b). By further rationalizing the reasons for the choice of a specific definition or concept view, authors can increase the comprehension of the reader of why a certain definition was chosen.

## Discussion

### Current State of Conceptual Ambiguity in Serious Games and Gamification Literature

Overall, the findings of our study help to better understand the phenomena of gamification and serious games and how they are conceptualized in research published in JMIR journals. Our results paint a heterogeneous landscape of different conceptualizations and different ways of distinguishing the 2 concepts. In addition, there are also some major differences in the evolution of definitions over time. We identified some common pitfalls that may arise when researchers deal with gamification or serious games and provide guidance on how to avoid them. In the following sections, we discuss the principal findings of this study.

First, the results from our literature review provide insights into the vast differences in how researchers conceptualize gamification or serious games. Our results show that less than half (121/265, 45.7%) of concept mentions are accompanied by an explicit definition which leaves room for improvements in scientific rigor. Regarding the sources used for definitions, our results show clear differences between gamification and serious games. Although the majority of definitions used for gamification (43/61, 70%) can be traced back to the seminal paper by Deterding et al [245], no such single central article exists for the concept of serious games. Regarding the content of definitions, in the case of gamification, most studies follow the understanding of Deterding et al [245]. Deviations from this understanding are particularly evident in a change of the *nongame context*, meaning that the context is either limited to a specific area of application (eg, health care and education) or omitted completely. From our perspective, both approaches can be problematic. First, we think that gamification and its potential to positively shape the cognition, affection, and behavior of people is not limited to a specific context. In fact, extant research has repeatedly stated that investigating the context specifics of gamification outcomes is one of the most interesting and challenging questions that researchers currently face [33,266]. Second, we think that omitting the *nongame context* from the definition of gamification can lead to great confusion around the concept, as this would technically allow including classic video games with pure entertainment purposes, which was actually intended to be avoided when conceptualizing gamification [245]. For serious games, the definition content was considerably more heterogeneous than that of gamification. However, one aspect stood out. Although some authors emphasize that serious games refer to video games in their entirety [85], others state that they only draw specific aspects from video games, such as design principles [88]. In the latter case, the definitions of serious games seemed considerably closer to the common definitions of gamification.

Second, by analyzing 59 studies that mentioned both gamification and serious games, our results reveal 4 approaches to the relationship between the 2 terms. From our point of view, 2 of these approaches, namely interchangeable use (5/59, 8%) and implicit differentiations (15/59, 25%), are not ideal for communicating one's own ideas of the concepts to the academic audience. As outlined in our reporting guidelines, we think that explicit definitions and distinctions play a key role in mitigating the conceptual ambiguity surrounding different types of game-based interventions. This is also echoed by extant literature on conceptual ambiguity problems, which states that such problems can arise when researchers implicitly assume that because they can make a logical distinction between concepts, this distinction will also exist in the minds of others [267]. The largest group of studies (22/59, 37%) labeled serious games as a type of gamified intervention or as the product resulting from the gamification process. This seems closely related to the classic process view of gamification [265], where gamification is construed as a process of transforming a purely utilitarian system into a system that combines utilitarian and hedonic functions by implementing game design elements [268]. From our point of view, this approach is incompatible with the understanding of the last group of studies (9/59, 15%) that, similar to Deterding et al [245], consolidate serious games as *full-fledged games*, whereas gamification involves systems that only partly consist of game design elements and in addition have nongame parts. A similar view is shared by Marczweski [259], who argues that contrary to gamification, serious games provide gameplay, which he describes as “[having] all the elements you would expect to see in a real game.” Although conceptually sound, this distinction can also be problematic, as deciding whether users actually *play* a game or *use* a system is an often complex question that involves empirical, subjective, and social factors [56,245]. In fact, for medical interventions in practice, it seems infeasible to empirically investigate each individual user on whether they are using or playing their system. On a conceptual level, this issue may be approached by substituting actual user behavior with design intention.

Finally, when analyzing the thematic focus of our review literature, our results show an ever-increasing amount of both gamification and serious games studies in the *JMIR* research community since 2015, with seemingly no concept prevailing over the other. We found this particularly interesting because it contrasts previous observations that gamification is increasingly superseding serious games as a more popular concept [18]. In fact, we also think that the circumstance that no concept prevails over the other even strengthens the relevance of clearly distinguishing between different types of game-based health interventions and attributing research results accurately in the future.

### Implications

Our study has several implications for future research. First, our study revealed a strong heterogeneity surrounding the conceptualizations of serious games and gamification. In particular, we demonstrate that just because 2 game-based interventions are labeled similarly, it does not necessarily follow that the designers share the same understanding of the underlying concept. For researchers, this implies that they need to exercise particular caution and scientific rigor when studying game-based interventions and reporting study results. In fact, the easy transferability of knowledge based on labels provided in the literature is often not given.

Second, our results strongly indicate that there are conceptual ambiguity problems surrounding serious games and gamification in the health care literature. To counteract this, we rigorously developed GAMING and evaluated them with the help of 9 outstanding experts from the area of game-based intervention research. To the best of our knowledge, this is the first study to formulate such reporting guidelines explicitly for game-based interventions. The individual guideline items are designed to mitigate common pitfalls that lead to conceptual ambiguities in the literature reviewed. Researchers can use GAMING as an inspiration for reporting the results of game-based health interventions while accounting for potential conceptual ambiguity. However, GAMING should not be interpreted as a prescription for the reporting of game-based health interventions in a strict or standardized format. The guideline items should be addressed in sufficient detail and with clarity somewhere in the manuscript, but the order and format for presenting the required information depends on author preferences, journal style, and the traditions of the research field.

For practitioners, we believe that our study can help raise awareness about the conceptual ambiguity surrounding game-based health interventions. Practitioners, similar to researchers, should exercise caution when interpreting research findings and transferring them into practice. Our review and guidelines may serve as a starting point to assess whether any study they want to draw on may be affected by conceptual ambiguity and subsequently support them in evaluating the transferability of knowledge for their own purposes.

### Limitations and Future Research

The results of our study are limited in several ways, which also opens up avenues for future research. First, we limited our literature review to studies published in the *Journal of Medical Internet Research* and its sister journals. Although we are convinced that the *Journal of Medical Internet Research* is a suitable representative of the overall landscape of game-based health interventions, future research may also benefit from investigating the conceptual ambiguities surrounding gamification and serious games outside of the *JMIR* research community. We would be particularly interested in whether our findings regarding the different evolutions of gamification and serious games definitions (Figures 2 and 3) hold in a different literature corpus. Researchers may also find it beneficial to extend our approach to a full citation network analysis to investigate the flow of knowledge in serious games and gamification literature [269]. This may allow further insights into whether given conceptualizations are refinements or extensions of existing ones, which is a prime indicator for investigating conceptual ambiguities [28]. Second, we only included two concepts in our literature review: gamification and serious games. As we alluded to in our introduction, a plethora of concepts for health interventions draws on games as their inspirational source. However, gamification and serious games are currently prevailing concepts in game-based interventions [13,18]. Thus, we argue that investigating the conceptual conflict between this particular concept pair allows for the extraction of the highest proportion of knowledge. Nevertheless, future research may still benefit from including additional or different concepts within the scope of the investigation. As for our reporting guidelines, we believe that we converted the insights from our literature review to a sufficient level of abstraction so that they are transferable to other types of game-based interventions.

### Conclusions

Games are an increasingly popular source of inspiration for the design of health care interventions. With the ever-increasing number of game-based interventions, concerns regarding conceptual ambiguity have arisen. In this study, we focus on the conceptual boundaries between two of the most prominent game-based intervention concepts: gamification and serious games. Our analysis of the literature in the *JMIR* research community unveils various understandings of the concepts themselves, as well as the boundaries between them. Thus, our results support the initial hypothesis of conceptual ambiguity between gamification and serious games in the health care literature. On the basis of these results, we proposed GAMING, consisting of 25 items designed to guide researchers in reporting their own game-based intervention studies in a way that mitigates conceptual ambiguity. We believe that our results can serve as a valuable supplement to existing research that has discussed conceptual differences between different types of game-based interventions and can help advance cumulative knowledge development in game-based health intervention research, without being impaired by conceptual ambiguity.

## Appendix

Multimedia Appendix 1 Overview of the 206 papers included in the literature review.

Multimedia Appendix 2 Consolidated, one-page version of Game-Based Intervention Reporting Guidelines, excluding rationales.

Multimedia Appendix 3 Consolidated, extended version of Game-Based Intervention Reporting Guidelines, including detailed rationales and exemplary statements.

Multimedia Appendix 4 Overview of existing distinctions between serious games and gamification in extant literature.

## Abbreviations

GAMING: Game-Based Intervention Reporting Guidelines
RQ: research question
