# Supplementary material for: Conceptual Ambiguity Surrounding Gamification and Serious Games in Health Care: Literature Review and Development of Game-Based Intervention Reporting Guidelines (GAMING)
Source: J Med Internet Res. 2021 Sep 10;23(9):e30390. doi: 10.2196/30390 (PMC8463952; doi:10.2196/30390)
Supplement: Multimedia Appendix 2 [file jmir_v23i9e30390_app2.docx]

| **#** | **Topic** | **Description** | **Exemplary / Explanatory Statements** |
| --- | --- | --- | --- |
| **1** | **Conceptual Focus** | (a) Decide which concepts^[[1]](#footnote-1)^ (ie gamification, serious games, ...) best reflect the interventions you want to investigate. | *-* |
|  |  | (b) Clearly state early in the paper which core concepts (ie gamification, serious games, ...) you focus on in your study, and why. | *“In this study, we focus on the concept of gamification, because we wanted to bring single game elements into the intervention instead of developing a full-fledged game.”* |
|  |  | (c) Supply only metadata (eg title, keywords) that corresponds to your core concepts. | *For a study developing a physical activity intervention based on gamification: Gamification, gamified intervention, …* |
| **2** | **Contribution** | (a) Decide which research stream^[[2]](#footnote-2)^ within the focused concepts your study contributes to. | *“Our work contributes to a better understanding of how gamification is being applied in real-world mHealth apps.“* |
|  |  | (b) Report which research streams your study contributes to and which criteria the decision for research streams was based on. | *“We contribute to a better understanding of the specific game element of leaderboards. We decided for leaderboards, as they are prominently used in mHealth apps to elicit social comparison.”* |
|  |  | (c) Clarify your study's contributions to the chosen research streams. | *“We provide rich insights into the psychological effects of leaderboards on patients when isolated from other game elements. […] Our insights are about leaderboard specifically and are not necessarily transferable to other social comparison features.”* |
|  |  | (d) Clarify your study's contributions to solving a problem or need in practice or society. | *“The results of our study can support the design and implementation of successful physical activity mHealth apps in practice.”* |
|  |  | (e) Report to which extent observed positive and negative outcomes can be attributed to your game-based approach. If possible, narrow down the attribution of outcomes based on individual components of your game-based approach (e.g., game elements). | *“Participants showed increased motivation, because they were able to compare themselves to others via the leaderboard function. However, this function also caused some participants to feel less competent, thus decreasing their motivation.”* |
| **3** | **Mindfulness about Related Concepts** | | |
| 3.1 | Introduction and Use of Related Concepts | (a) Make efforts to identify possibly related concepts prominent in the context of your study. | *Prominent related concepts for gamification in physical activity: Exergames, active video games, fitness games, ...* |
|  |  | (b) Mention only those related concepts that are substantive for your study. | *‘Substantive’ in the sense that a research design necessitates the introduction of a concept. Example: A research design contrasting the effects of two game-based intervention concepts requires the introduction of both concepts.* |
|  |  | (c) Be mindful about nuanced terms in the domain of any introduced concept and use established vocabulary precisely. | *Examples: Game design elements, game mechanics, gamification elements, ...* |
|  |  | (d) Avoid using related concepts interchangeably. If you use an umbrella term, specify which terms it comprises, and clarify why you introduce it. | *“To allow a better readability of the manuscript, we use the term activity games to describe gamified physical activity interventions, serious games for physical activity, as well as exergames.”* |
| 3.2 | Insights from Extant Literature | (a) Be mindful about conceptual ambiguities when drawing on literature about game-based interventions. | *-* |
|  |  | (b) Do not presume easy transferability of insights from one concept to another. | *Example: Drawing on serious games literature for a gamification-based intervention (or vice versa).* |
|  |  | (c) Specify precisely what you draw from literature and why these insights are applicable to your study. | *“Serious games and gamification share that they both center around game elements. Hence, to compile a list of possible game elements for our gamified intervention, we also drew upon serious games literature to widen our scope.”* |
| **4** | **Individual Concept Definitions** | | |
| 4.1 | Definition Inspiration | (a) Familiarize yourself with definitions for a concept provided by extant literature. | *Reviews can often provide a good overview of different views on a concept.* |
|  |  | (b) Decide, whether a concept definition from extant literature is applicable for your research, or if you need a self-developed definition. | *Decision Criteria: Deficits in extant literature? Incompatibility of own views with literature?* |
| 4.2 | Definition of Concepts | (a) Explicitly define each introduced concept independently in a principal clause. | *“We define gamification as the use of game design elements in non-game contexts. We define serious games as games whose primary purpose is not entertainment. A game is [...].”* |
|  |  | (b) Explicitly distinguish each introduced related concept pairwise to at least to your core concepts; better even to all related concepts. | *Gamification differs from serious games in that [...].* |
| 4.3 | Definitions from Extant Literature | If definitions are taken from extant literature… |  |
|  |  | (a) Make efforts to identify the original source of a definition. | *-* |
|  |  | (b) Include an explicit reference to the source of a definition directly following the definition. | *“We define gamification as the use of game-design elements in non-game contexts (Deterding et al., 2011).”* |
| 4.4 | Self-Developed Definitions | If any definition for a concept is self-developed... |  |
|  |  | (a) Make sure to adhere to good definition design. | *Be specific, avoid long sentences, do not repeat the term to be defined in the definition, ...* |
|  |  | (b) Clarify from which views your self-developed definition emerged. | *“We include only specific game elements of point, badges and leaderboards in our definition of gamification, because [...].”* |
| 4.5 | Multiple Definitions for a Single Concept | If multiple definitions for a single concept are provided … | *“Gamification can be either defined as [...][1] or as [...][2].”* |
|  |  | (a) state clearly, which definitions are applied in the study, and why. | *“For the purposes of this study, we follow the view of [2], because [...].”* |
|  |  | (b) apply the chosen definition(s) consistently. | *-* |

1. In the context of our guidelines, concept refers to a class of game-based interventions (e.g., gamification, serious games, exergames) [↑](#footnote-ref-1)
2. In the context of our guidelines, a research stream refers to a set of studies with similar properties, such as their research method, or their healthcare context [↑](#footnote-ref-2)
