# Supplementary material for: Conceptual Ambiguity Surrounding Gamification and Serious Games in Health Care: Literature Review and Development of Game-Based Intervention Reporting Guidelines (GAMING)
Source: J Med Internet Res. 2021 Sep 10;23(9):e30390. doi: 10.2196/30390 (PMC8463952; doi:10.2196/30390)
Supplement: Multimedia Appendix 3 [file jmir_v23i9e30390_app3.docx]

Table MA3-1. Game-based Intervention Reporting Guidelines (GAMING) Items

| **#** | **Topic** | **Description** | **Rationale** | **Exemplary / Explanatory Statements** |
| --- | --- | --- | --- | --- |
| **1** | **Conceptual Focus** | (a) Decide which concepts (ie gamification, serious games, ...) best reflect the interventions you want to investigate. | *To be able to report the conceptual focus of their study, authors first have to figure out themselves, which concept they are following, and based in which criteria they decide for a concept. Ideally, the conceptual focus of a study should already be established in the ideation phase of a research project.* | *-* |
|  |  | (b) Clearly state early in the paper which core concepts (ie gamification, serious games, ...) you focus on in your study, and why. | *Allows readers to immediately grasp a study’s conceptual focus.* | *“In this study, we focus on the concept of gamification, because we wanted to bring single game elements into the intervention instead of developing a full-fledged game.”* |
|  |  | (c) Supply only metadata (eg title, keywords) that corresponds to your core concepts. | *Many researchers also use metadata or early sections of a paper to initially assess the relevance of a study for their purposes. Metadata related to non-core concepts may thus alter other researchers' perception of whether a manuscript is part of a research stream or not.*  *For meta-studies, broadly diversified metadata may also increase noise.* | *For a study developing a physical activity intervention based on gamification: Gamification, gamified intervention, …* |
| **2** | **Contribution** | (a) Decide which research stream within the focused concepts your study contributes to. | *There are various research streams proximal to different concepts, each with their own thematic emphasis and viewing angle. Hence, transferability of knowledge may be limited. Accordingly, choosing a research stream to contribute should be a concious and careful decision.* | *“Our work contributes to a better*  *understanding of how gamification is being applied in real-world mHealth apps.“* |
|  |  | (b) Report which research streams your study contributes to and which criteria the decision for research streams was based on. | *Allows readers a better understanding of the setting of a study and enables readers to better gauge the transferability as well as generalizability of a study's results.* | *“We contribute to a better understanding of the specific game element of leaderboards. We decided for leaderboards, as they are prominently used in mHealth apps to elicit social comparison.”* |
|  |  | (c) Clarify your study's contributions to the chosen research streams. | *Allows readers to more easily understand, how a study contributes to a better understanding of the core concepts.* | *“We provide rich insights into the psychological effects of leaderboards on patients when isolated from other game elements. […] Our insights are about leaderboard specifically and are not necessarily transferable to other social comparison features.”* |
|  |  | (d) Clarify your study's contributions to solving a problem or need in practice or society. | *The ultimate goal of an intervention is usually to solve a problem in pratice or society (eg sedentary lifestyles). Accordingly, for an understanding of how specific game-based approaches can solve these problems, an intervention’s practical contribution should be clarified.* | *“The results of our study can support the design and implementation of successful physical activity mHealth apps in practice.”* |
|  |  | (e) Report to which extent observed positive and negative outcomes can be attributed to your game-based approach. If possible, narrow down the attribution of outcomes based on individual components of your game-based approach (e.g., game elements). | *For an understanding of game-based approaches, it is vital that intervention outcomes (both positive and negative) are attributed to the chosen game-based approach as granularly as possible. This allows readers to easily contextualize knowledge and understand the attribution of outcomes to for example specific game elements (eg leaderboards) or affective outcomes (eg envy).* | *“Participants showed increased motivation, because they were able to compare themselves to others via the leaderboard function. However, this function also caused some participants to feel less competent, thus decreasing their motivation.”* |

Table MA3-1. Game-based Intervention Reporting Guidelines (GAMING) Items (continued)

| **#** | **Topic** | **Description** | **Rationale** | **Exemplary / Explanatory Statements** |
| --- | --- | --- | --- | --- |
| **3** | **Mindfulness about Related Concepts** | | | |
| 3.1 | Introduction and Use of Related Concepts | (a) Make efforts to identify possibly related concepts prominent in the context of your study. | *The proximity of two game-based approaches can vary based on context. Some game-based approaches are more prominent in certain contexts than others (e.g., exergames in the context of physical activity interventions). Hence, when operating within a specific context, researchers should aim to identify concepts with a high 'contextual proximity', as it is the boundaries to these concepts that are most important to establish to avoid conceptual ambiguity.* | *Prominent related concepts for gamification in physical activity: Exergames, active video games, fitness games, ...* |
|  |  | (b) Mention only those related concepts that are substantive for your study. | *Increasing the number of introduced concept produces more potential pairwise conceptual ambiguities. Hence, authors should carefully consider the benefits of introducing related concepts, and only do so if it is substantive for their study design.* | *‘Substantive’ in the sense that a research design necessitates the introduction of a concept. Example: A research design contrasting the effects of two game-based intervention concepts requires the introduction of both concepts.* |
|  |  | (c) Be mindful about nuanced terms in the domain of any introduced concept and use established vocabulary precisely. | *In some areas, specific terms are possibly very nuanced and thus may not necessarily correspond to their intuitive meaning. Similarly, some terms describe very specific circumstances, and should thus not be used interchangeably with ostensibly synonymous terms (e.g., 'game design principles' instead of 'game design elements').* | *Examples: Game design elements, game mechanics, gamification elements, ...* |
|  |  | (d) Avoid using related concepts interchangeably. If you use an umbrella term, specify which terms it comprises, and clarify why you introduce it. | *An interchangeable use of concepts may easily be interpreted as there being no theoretical or empirical differences between the corresponding concepts. However, there are at least theoretical differences between different concepts.* | *“To allow a better readability of the manuscript, we use the term activity games to describe gamified physical activity interventions, serious games for physical activity, as well as exergames.”* |
| 3.2 | Insights from Extant Literature | (a) Be mindful about conceptual ambiguities when drawing on literature about game-based interventions. | *For each concept, there may be various, possibly not reoncilable conceptualizations. Hence, authors should always challenge the label of a game-based intervention against their own views of a respective concept, instead of immediately assuming the relevance of a study based on its label.* | *-* |
|  |  | (b) Do not presume easy transferability of insights from one concept to another. | *Theoretical and empirical differences between concepts can easily hamper the transferability of knowledge.* | *Example: Drawing on serious games literature for a gamification-based intervention (or vice versa).* |
|  |  | (c) Specify precisely what you draw from literature and why these insights are applicable to your study. | *Allows the reader a better understanding of how knowledge from literature is transferable, despite possible theoretical or empirical differences.* | *“Serious games and gamification share that they both center around game elements. Hence, to compile a list of possible game elements for our gamified intervention, we also drew upon serious games literature to widen our scope.”* |

Table MA3-1. Game-based Intervention Reporting Guidelines (GAMING) Items (continued)

| **#** | **Topic** | **Description** | **Rationale** | **Exemplary / Explanatory Statements** |
| --- | --- | --- | --- | --- |
| **4** | **Individual Concept Definitions** | | | |
| 4.1 | Definition Inspiration | (a) Familiarize yourself with definitions for a concept provided by extant literature. | *For most concepts, multiple prominent definitions with various viewpoints exist.*  *Familiarizing themselves with these definitions can increase author’s understanding of a concept.* | *Reviews can often provide a good overview of different views on a concept.* |
|  |  | (b) Decide, whether a concept definition from extant literature is applicable for your research, or if you need a self-developed definition. | *Establishing and advancing concept definitions is one of the most tedious, long-winded steps in any research stream, but also usually one that usually brings vast benefits about theoretical advancement. Thus, we do not want to discourage authors to develop their own novel definition for a concept in order to represent their own, possibly fruitful viewpoint. However, we also urge authors not to 'reinvent the wheel', and instead fall back on established definitions wherever possible, as this allows a better placement of a study within extant research and facilitate the building of cumulative knowledge.* | *Decision Criteria: Deficits in extant literature? Incompatibility of own views with literature?* |
| 4.2 | Definition of Concepts | (a) Explicitly define each introduced concept independently in a principal clause. | *A lack of an explicit definition for a concept requires the reader to subjectively appraise the authors’ understanding of it based on context. This appraisal is highly error-prone. Explicit definitions alleviate the need for such appraisals.* | *“We define gamification as the use of game design elements in non-game contexts. We define serious games as games whose primary purpose is not entertainment. A game is [...].”* |
|  |  | (b) Explicitly distinguish each introduced related concept pairwise to at least to your core concepts; better even to all related concepts. | *Explicitly drawing the line between concepts is the most reliable way to mitigate conceptual ambiguity. Ideally, whenever feasible, the boundaries between all possible pairs of introduced concepts are drawn.* | *Gamification differs from serious games in that [...].* |
| 4.3 | Definitions from Extant Literature | If definitions are taken from extant literature… |  |  |
|  |  | (a) Make efforts to identify the original source of a definition. | *Most research streams surrounding game-based concepts have seminal papers, to which term definitions can often be traced back. Experienced reserachers are usually able to recognize these seminal papers just from their in-text reference and can thus immediately attribute a given definition to its corresponding definition stream. Indirect citations only obfuscate the origin of a definition, as they require the reader to 'jump through hoops' to find the original source of a definition.* | *-* |
|  |  | (b) Include an explicit reference to the source of a definition directly following the definition. | *Further ensures that a definition can be attributed to its source.* | *“We define gamification as the use of game-design elements in non-game contexts (Deterding et al., 2011).”* |
| 4.4 | Self-Developed Definitions | If any definition for a concept is self-developed... |  |  |
|  |  | (a) Make sure to adhere to good definition design. | *Formulating definitions is an arduous yet crucial task. Good definition design ensures that the meaning of a concept is successfully conveyed to the reader.* | *Be specific, avoid long sentences, do not repeat the term to be defined in the definition, ...* |
|  |  | (b) Clarify from which views your self-developed definition emerged. | *Fosters readers’ understanding of the decision for a self-developed definition and thus allows the readera better grasp of how a self-developed definition contributes an advanced, possibly novel understanding of a concept.* | *“We include only specific game elements of point, badges and leaderboards in our definition of gamification, because [...].”* |
| 4.5 | Multiple Definitions for a Single Concept | If multiple definitions for a single concept are provided … |  | *“Gamification can be either defined as [...][1] or as [...][2].”* |
|  |  | (a) state clearly, which definitions are applied in the study, and why. | *Providing multiple definitions for a concept can be beneficial to contrast different views. However, it can also quickly become confusing for the reader, when he or she does not know which definitions authors apply in their study. This confusion may be exacerbated when several definitions are not reconcilable.* | *“For the purposes of this study, we follow the view of [2], because [...].”* |
|  |  | (b) apply the chosen definition(s) consistently. | *Possibly leads to confusion in readers if they cannot reconcile a statement with the definitions stated as chosen.* | *-* |
