# Supplementary material for: Conceptual Ambiguity Surrounding Gamification and Serious Games in Health Care: Literature Review and Development of Game-Based Intervention Reporting Guidelines (GAMING)
Source: J Med Internet Res. 2021 Sep 10;23(9):e30390. doi: 10.2196/30390 (PMC8463952; doi:10.2196/30390)
Supplement: Multimedia Appendix 4 [file jmir_v23i9e30390_app4.docx]

## Multimedia Appendix 4. Extant distinctions between gamification and serious games.

In extant literature, various attempts to disambiguate the concepts of gamification and serious games can be found. As most of these distinctions make certain assumptions or are restricted to a specific context, no single distinction is applicable for every study. Therefore, we compiled a list of distinctions between gamification and serious games from extant literature, which can be found in Table MA1-1.

Table MA4-1. Extant distinctions between gamification and serious games.

| **Study** | **Main Questions** | **Distinction Criteria** | | **Assumption** |
| --- | --- | --- | --- | --- |
|  |  | **Gamification** | **Serious Game** |  |
| Deterding et al. (2011) | Is it a full-fledged game for non-entertainment purposes or does it only contain game design elements? | Only contains game design elements | Full-fledged game for non-entertainment purposes | Game design elements are characteristic to games (i.e. are found in most games, are readily associated with games, and play a significant role in gameplay) |
| McCallum (2012) | Is it an addition of game elements to an existing activity or does it require creating a whole new experience to engage the user? | Addition of game elements to an existing activity | Creation of a whole new experience to achieve some change in the player | The distinction between the approaches refers to the way in which the experience has been designed |
| Marczewski (2013) | Does it play like a game? Does it provide true gameplay? | Does not play like a game; no true gameplay | Plays like a game; true gameplay | Both approaches have a main purpose other than entertainment; no clear definition of what constitutes ‘true gameplay’ |

Table MA4-1. Extant distinctions between gamification and serious games. (continued)

| **Study** | **Main Questions** | **Distinction Criteria** | | **Assumption** |
| --- | --- | --- | --- | --- |
|  |  | **Gamification** | **Serious Game** |  |
| Landers (2014) | Does it affect learning directly or indirectly? Does it have all or only specific game attributes? | Affects contextual learner behavior or attitude; particular game attributes | Affects learning directly; all game attributes, but to varying degrees | Pre-existing learning process |
| Yohannis et al. (2014) | Is it presented as a game (does it have all the game characteristics)? | Not presented as a game; does not have all the game characteristics | Presented as a game; does have all the game characteristics | Both approaches are applied to a gameless object;  the final product of gamification can be a serious game |
| Santhanam et al. (2015) | Is it an additional game layer of a non-gaming task or is it a separate full-fledged game for learning? | Additional game layer of a non-gaming task | Separate full-fledged game for learning | Gamification employs an additional game layer, which is usually easy to distinguish |
| Liu et al. (2017) | Does the real-world system sacrifice some or all of its functionality? Is the approach incorporated into the real-world system or used separately from it? | Does not sacrifice functionality; incorporated into real-world system | Sacrifices functionality; used separately from real-world system | Pre-existing real-world system, to which game elements can be added or after which new systems can be modeled |
| De Croon et al. (2018) | Is it a complete game? Does it provide game play? | Game design elements in a non-gaming context; does not provide game play | Complete game with all game elements; provides game play | Both approaches try to motivate the user, but are not the same |

## References

[1] S. Deterding, D. Dixon, R. Khaled, and L. Nacke, “From game design elements to gamefulness: defining "gamification",” in Proceedings of the 15th International Academic MindTrek Conference Envisioning Future Media Environments, Tampere, Finland, 2011, p. 9.

[2] S. McCallum, “Gamification and serious games for personalized health,” in pHealth, 2012, pp. 85–96.

[3] Andrej Marczweski, What’s the difference between Gamification and Serious Games? [Online]. Available: https://www.gamasutra.com/blogs/AndrzejMarczewski/20130311/188218/Whats_the_difference_between_Gamification_and_Serious_Games.php (accessed: Apr. 20 2021).

[4] R. N. Landers, “Developing a Theory of Gamified Learning,” Simulation & Gaming, vol. 45, no. 6, pp. 752–768, 2014, doi: 10.1177/1046878114563660.

[5] A. R. Yohannis, Y. Denny Prabowo, and A. Waworuntu, “Defining gamification: From lexical meaning and process viewpoint towards a gameful reality,” in 2014 International Conference on Information Technology Systems and Innovation (ICITSI), Bandung, Indonesia, Nov. 2014 - Nov. 2014, pp. 284–289.

[6] R. Santhanam, de Liu, and W.-C. M. Shen, “Research Note—Gamification of technology-mediated training: Not all competitions are the same,” Information systems research, vol. 27, no. 2, pp. 453–465, 2016.

[7] de Liu, R. Santhanam, and J. Webster, “Toward Meaningful Engagement: A Framework for Design and Research of Gamified Information Systems,” MIS quarterly, vol. 41, no. 4, 2017.

[8] R. de Croon, D. Wildemeersch, J. Wille, K. Verbert, and V. V. Abeele, “Gamification and serious games in a healthcare informatics context,” in 2018 IEEE International Conference on Healthcare Informatics (ICHI), 2018, pp. 53–63.
